# P-1850. Acute *Babesia microti* Infection in Humans Is Associated With Marked Changes In The Expression Of Peripheral Blood Coding And Noncoding RNA

**DOI:** 10.1093/ofid/ofae631.2011

**Published:** 2025-01-29

**Authors:** Dana Mordue, Paul Arnaboldi, David Carlson, Pooja Lamba, Rudline G Zamor, Victoria A Bateman, Eric Spitzer, Luis A Marcos

**Affiliations:** New York Medical College, Valhalla, New York; New York Medical College, Valhalla, New York; Stony Brook University, Stony Brook, New York; Stony Brook University, Stony Brook, New York; Stony Brook University Hospital, Stony Brook, New York; Stony Brook University, Stony Brook, New York; Stony Brook Medicine, Stony Brook, New York; Stony Brook University Hospital, Stony Brook, New York

## Abstract

**Background:**

Babesiosis is a potentially life-threatening disease transmitted by ticks. However, host and parasite determinants of human babesiosis are completely unknown. In the current study we evaluated the impact of *B. microti* infection, the primary cause of human babesiosis in the US, on the peripheral blood transcriptome.

**Figure 1.**
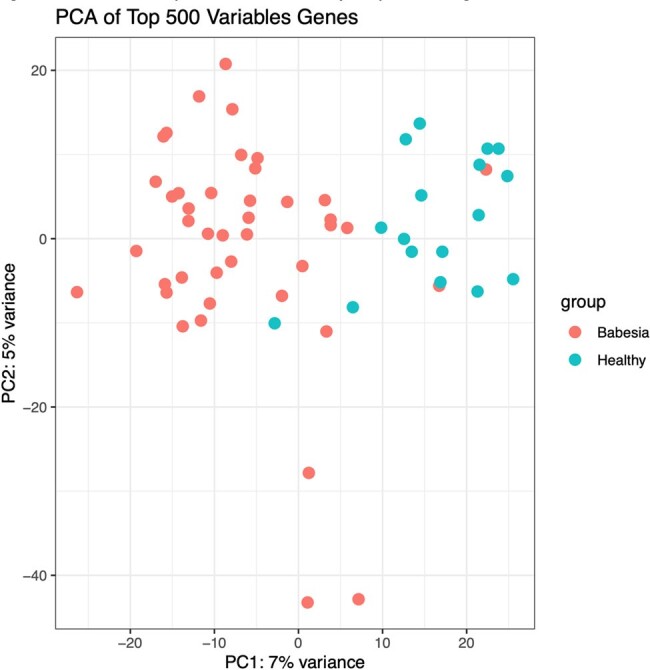
PCA of top 500 differentially expressed genes.

PCA distinguishes Babesia patients at presentation from healthy controls.

**Methods:**

Patients with confirmed *B. microti* acute infection were enrolled into a 1-year follow up cohort study at Stony Brook University Hospital, NY. Total RNA was isolated from blood using PaxGene RNA tubes and library preparation performed using Illumina's Stranded Total RNAPrep with Ribo-zero plus kit to remove human ribosomal RNA and globin RNA. 100 M RNA paired end reads were sequenced (2 x 151 bps). Low expression RNAs with less than 10 reads in 17 samples were filtered out prior to identification of differentially expressed genes (DEGs) (p< 0.05).

**Figure 2.**
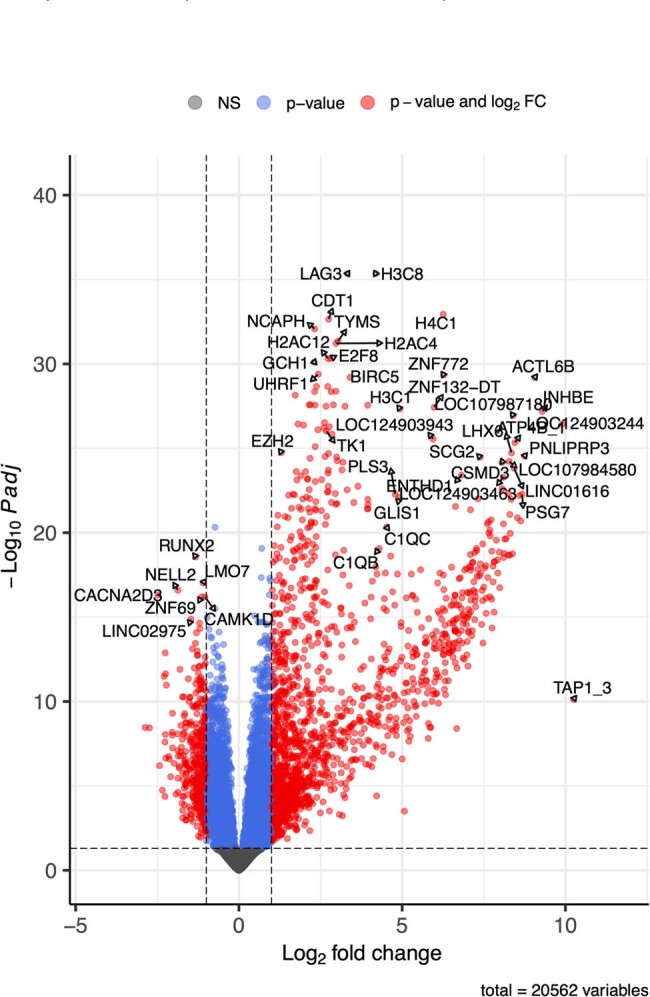
Volcano Plot comparing differential gene and noncoding RNA expression between healthy controls and patients with babesiosis at presentation. Red circles represent genes whose expression are increased or decreased in patients with babesiosis at presentation compared to healthy controls. Blue circles represent genes whose expression are not significantly different.

**Results:**

The peripheral blood transcriptome from 20 healthy controls was compared to 42 patients at presentation for *B. microti* infection and 1, 3, 6, 9 and 12 months after treatment for patients who completed the longitudinal protocol after the first visit. A total of 1860 genes or noncoding RNAs were differentially expressed in patients with babesiosis at presentation (V0) compared to healthy controls; 370 were decreased in patients with babesiosis and 1490 were increased (adj p < 0.01; expression fold change >+/- 1.) PCA analysis (Fig 1), and the Volcano Plot (Fig. 2) demonstrate that patients with babesiosis have a distinct peripheral blood gene expression profile compared to healthy control individuals. The heat map of DEGs in Fig. 3 shows the peripheral blood transcriptome in patients with babesiosis largely returned to baseline levels by 1-month post-treatment. The Qiagen IPA Graphical Summary based on DEGs is shown in Figure 4 and highlights activation of diverse immune-mediators, including TNF and interferon families, as well as growth factors, and vascularization pathways in response to *B. microti* infection.

**Figure 3.**
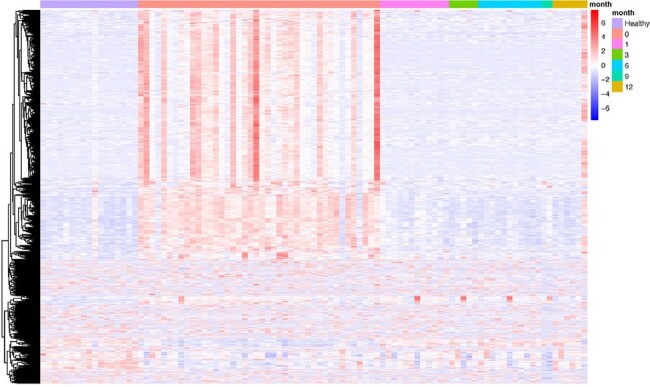
Heat map of differentially expressed genes of patients with babesiosis at presentation (0) and 1, 3, 6, 9 and 12 months post-treatment compared to healthy control individuals. Gene expression in patients with babesiosis is markedly different at presentation but is similar to levels for healthy controls by 1 month post-treatment.

**Conclusion:**

Infection with *B. microti* results in marked changes in expression of genes and noncoding RNA in peripheral blood that largely returns to baseline by 1-month post-treatment.

**Figure 4:**
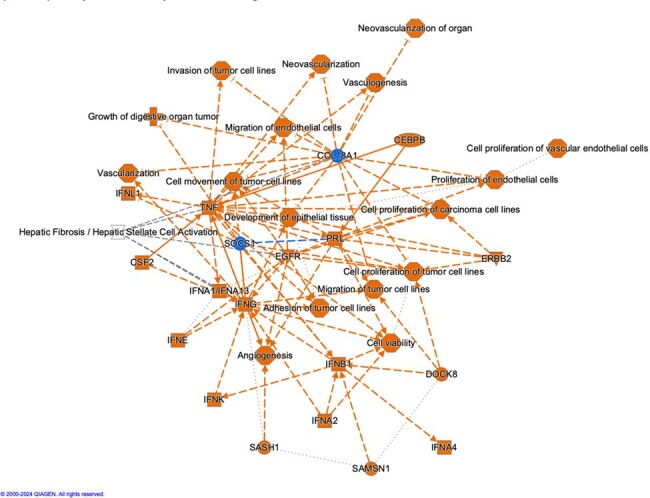
Qiagen IPA Graphical Summary of pathways likely increased (orange) or decreased (blue) in patients presenting with babesiosis.

**Disclosures:**

**Paul Arnaboldi, PhD**, Biopeptides, Corp: Patent Holder|Biopeptides, Corp: Employee|DiaSorin: Advisor/Consultant

